# A novel study on amyloid β peptide 40, 42 and 40/42 ratio in Saudi autistics

**DOI:** 10.1186/1744-9081-8-4

**Published:** 2012-01-13

**Authors:** Laila Y Al- Ayadhi, Abir G Ben Bacha, Malak Kotb, Afaf K El-Ansary

**Affiliations:** 1 Department of Physiology, Faculty of Medicine, King Saud University, Riyadh, Saudi Arabia; 2 Autism Research and Treatment Center, Riyadh, Saudi Arabia; 3 Shaik AL-Amodi Autism Research Chair, King Saud University, Riyadh, Saudi Arabia; 4 Biochemistry Department, Science College, King Saud University, Riyadh, Saudi Arabia; 5 Department of Molecular Genetics, Biochemistry and Microbiology, College of Medicine, University of Cincinnati, Cincinnati, Ohio, USA

**Keywords:** Autism, Neurotoxicity, Amyloid beta, Brain influx, Cognitive disability

## Abstract

**Objectives:**

We examined whether plasma concentrations of amyloid beta (Aβ) as protein derivatives play a central role in the etiology of autistic features.

**Design and Methods:**

Concentrations of human Aβ (1-42), Aβ (1-40), and Aβ (40/42) in the plasma of 52 autistic children (aged 3-16 years) and 36 age-matched control subjects were determined by using the ELISA technique and were compared.

**Results:**

Compared to control subjects, autistic children exhibited significantly lower concentrations of both Aβ (1-40) and Aβ (1-42) and lower Aβ (40/42) concentration ratio. Receiver operating characteristics curve (ROC) analysis showed that these measurements of Aβ peptides showed high specificity and sensitivity in distinguishing autistic children from control subjects.

**Conclusions:**

Lower concentrations of Aβ (1-42) and Aβ (1-40) were attributed to loss of Aβ equilibrium between the brain and blood, an imbalance that may lead to failure to draw Aβ from the brain and/or impairment of β- and γ- secretase's concentration or kinetics as enzymes involving in Aβ production.

## 1. Introduction

Autism and other related autism spectrum disorders (ASDs) are behavioral syndromes that include various degrees of verbal, nonverbal, and social impairment, as well as restricted or stereotyped interests and activities. The disorders are characterized by early onset (before 36 months of age) [1,2] and by long-lasting social or cognitive handicaps. With an overall prevalence of approximately 0.6% [3], ASDs are an important public health problem worldwide. Although international consensus considers these syndromes to be phenotypic expressions of impairments affecting the development of the central nervous system (CNS), numerous questions concerning their etiopathology are still unanswered.

Children with autism generally find it difficult to ignore irrelevant information and are easily distracted by other stimuli. Therefore, we can assume that these children may have a selective attention deficit. In humans, prenatal stress is linked to an increased vulnerability to various psychosocial problems of childhood and adulthood. In children, stress is associated with cognitive, behavioral, physical, and emotional problems [4-7], as well as with autism [8-10].

Free radicals seem to be implicated in the onset of autism. Reactive oxygen species (ROS), including superoxide (O_2_^-^), hydroxyl (^_^OH), hydrogen peroxide (H_2_O_2_), singlet oxygen (^1^O_2_), and nitric oxide (NO^_^), are produced through physiologic and pathologic processes [11]. ROS are scavenged by specific defense systems, including antioxidant enzymes (superoxide dismutase [SOD], catalase [CAT], glutathione peroxidase [GPx]) and nonenzymatic antioxidants such as glutathione (GSH) and metallothioneins (MTs). Many autistic children seem to share a chronic flaw in the defense systems against ROS. In studies of the RBC of autistic children, Sogut et al. (2003) found higher concentrations of NO^_ ^and GPx [12], Zoroglu et al. (2004) reported higher concentrations of NO^_ ^and thiobarbituric acid-reactive substances (TBARs) [13], Chauhan et al. (2004) found a reduction in antioxidant proteins [14], and Geier et al. (2009) and Al-Gadani et al. (2009) described a decrease in reduced GSH [15,16]. In autistic Saudi children, overexpression of SOD, together with slightly inhibited CAT activity, indicated that these children are under H_2_O_2 _stress [16]. It is well known that glutamate is inhibited by astrocytes in a concentration-dependent manner. The inhibition of CAT clearly potentiated this effect.

Alzheimer's disease (AD), the primary dementing disorder of the elderly, affects more than four million persons in the United States. Aging is the chief risk factor for AD. Important pathological hallmarks of AD include loss of synapses and the presence of senile plaques (SPs) and neurofibrillary tangles (NFTs). SPs consist of a highly dense core of Aβ peptide, a peptide 39 to 43 amino acids in length (1-42) that is surrounded by dystrophic neurites [17]. Aβ (1-40), which composes approximately 90% of total secreted Aβ, aggregates much more slowly than Aβ (1-42) [18]. Aβ in amyloid plaques consists mainly of the Aβ (1-42) species, whereas vascular amyloid is composed primarily of Aβ (1-40). The relatively high solubility of Aβ (1-40) may allow this species to diffuse for greater distances than the less soluble Aβ (1-42), thereby increasing its deposition around brain vessels [19].

A growing body of evidence indicates that Aβ peptide toxicity is mediated by free radical damage to cell membranes [20-23]. The concept that Aβ induces lipid peroxidation is a key component of the Aβ-associated free radical model of neurodegeneration in AD [23,24]. Consistent with a free radical process, Aβ causes lipid peroxidation in brain cell membranes, and this peroxidation is inhibited by free radical antioxidants [21,23]. Giedraitis et al. (2007), suggest that the normal equilibrium between cerebrospinal fluid (CSF) and plasma Aβ may be disrupted in AD patients and may result in the initiation of amyloid deposition in the brain [25].

The findings of in vitro studies of lipid peroxidation induced by Aβ (1-42) and postmortem studies of lipid peroxidation (and its sequelae) in the AD brain, together with the confirmed role of oxidative stress in the etiology of autism [14-16], initiated our interest to study plasma concentration of Aβ peptide in autistic Saudi children and age-matched control subjects in an attempt to investigate the equilibrium status between the brain and blood and to highlight other factors that might contribute in the alteration of plasma Aβ peptide concentration. This comparison may help to clarify the causative role of Aβ peptide-induced oxidative stress in the pathology of autism and the possibility to use both as biomarkers of this disorder if they recorded remarkable sensitivity and specificity upon performing receiver operating characteristics statistical analysis. This could help in the early diagnosis and intervention to control the prevalence of this disease.

## 2. Materials and methods

### 2.1. Subjects and methods

The study protocol followed the ethical guidelines of the most recent Declaration of Helsinki (Edinburgh, 2000). Written informed consent was provided by the children's parents, and the children themselves assented to participation if they were developmentally able to do so. Subjects for this study were enrolled through the Autism Research and Treatment (ART) Center clinic, whose sample population consists of children aged 3 to 16 years with a diagnosis of ASD. The diagnosis was confirmed by using the Autism Diagnostic Interview-Revised (ADI-R), the Autism Diagnostic Observation Schedule (ADOS), and the Developmental, Dimensional Diagnostic Interview (3DI). Of the 52 autistic children, 40 were nonverbal and 12 were verbal. The intelligence quotient (IQ) of all autistic children was lower than 80. All children had sporadic autism (simplex cases), and all tested negative for the Fragile × syndrome. The control subjects were recruited from the well-baby clinic at King Khaled University Hospital; they also ranged in age from 3 to 16 years. Subjects were excluded from the study if they had dysmorphic features, tuberous sclerosis, Angelman syndrome, or other serious neurological (e.g., seizures), psychiatric (e.g., bipolar disorder), or medical (e.g., endocrine, cardiovascular, pulmonary, liver, kidney) conditions. All participants were screened via parental interview for current and past physical illness.

### 2.2. Samples collection

After overnight fast, 10 ml blood samples were collected from both groups in test tubes containing sodium heparin as anticoagulant. Tubes were centrifuged at 3500 rpm at room temperature for 15 minutes, plasma was obtained and deep freezed (at -80°C) until analysis time.

### 2.3. Measurement of Aβ (1-40) and Aβ (1-42)

Plasma concentrations of Aβ were measured by using the human Aβ (1-40) and Aβ (1-42) TGC ELISA kit (The Genetics Company, Schlieren, Switzerland) according to the manufacturer's instructions. Briefly, plasma samples were 100 times diluted in assay buffer and processed according to the manufacturer's recommended protocols. Samples and standards were incubated in capture wells overnight at 8°C with antibodies specific for Aβ (1-40) or Aβ (1-42). The capture antibody was 6E10 (Sigma, St Louis, Missouri), and the detection antibody was a biotin-labelled G2-10 (The Genetics Company, Schlieren, Switzerland). The synthetic Aβ (1-40) peptide (Bachem, Bubendorf, Switzerland) was used as the standard. After several rinses, the enzyme-conjugated detection reagent was added to the wells for 30 minutes. After additional rinses, wells were incubated with the chromogen solution for 30 minutes at room temperature, shielded from light. After the addition of the stop solution, the wells were read for absorption at 450 nm, and the Aβ concentration in the samples was calculated from standard curves. The detection limit was 25 pg/mL.

### 2.4. Statistical analysis

Results were expressed as means ± S.D. Statistical comparisons were performed with independent t-tests with the Statistical Package for the Social Sciences (SPSS). Significance was assigned at the level of P < 0.05. Receiver operating characteristics curve (ROC) analysis was performed. Area under the curve, cutoff values, and degree of specificity and sensitivity were calculated.

## 3. Results

**Figure 1:**
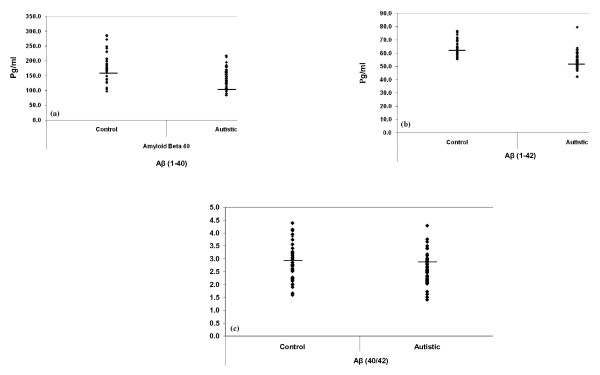
**Levels of the measured parameters in plasma.** Mean of measured Aβ (1-40) (a), Aβ (1-42) (b), and Aβ (40/42) (c) in autistic patients (N=52) compared to age- matching controls (N=36). Mean value for each group is designated by a line. Aβ level is expressed as pg/mL plasma.

Table 1 presents plasma concentrations of Aβ (1-40), Aβ (1-42), and Aβ (40/42) ratio. Compared to age-matched control subjects, autistic children exhibited significantly lower plasma concentrations of Aβ (1-40) and Aβ (1-42) (P < 0.05) and non-significant lower Aβ (40/42) ratio (P = 0.168). Figure 1 illustrates the mean values of the measured Aβ peptides. The figures clearly show that overlap in the distributed values around the means of the autistic and control groups was seen in the concentrations of Aβ (1-40). This overlap could be due to the fact that the individual data set within each group was dispersed or spread out around the means.

**Table 1:** Mean ± S.D of plasma levels of Aβ (1-40), Aβ (1-42) and Aβ (40/42) ratio in autistic patients (N = 52) compared to age- matching controls (N = 36).

Parameter	Group	N	Minimum	Maximum	Mean ± S.D	P value
Aβ (1-40)	Control	36	96.45	285.97	185.43 ± 48.76	0.000
		
	Autistic	52	84.03	217.1	145.07 ± 28.72	

Aβ (1-42)	Control	36	55.38	76.54	64.72 ± 5.67	0.000
		
	Autistic	51	42.12	79.62	54.86 ± 6.16	

Aβ (40/42)	Control	36	1.59	4.39	2.86 ± 0.71	0.168
		
	Autistic	50	1.41	4.29	2.67 ± 0.58	

This table describes the independent t-test between the control and autistic groups regarding levels of Aβ (1-40) andAβ (1-42) expressed as pg/mL plasma and Aβ (40/42) ratio. Significant level at p < 0.05.

**Figure 2:**
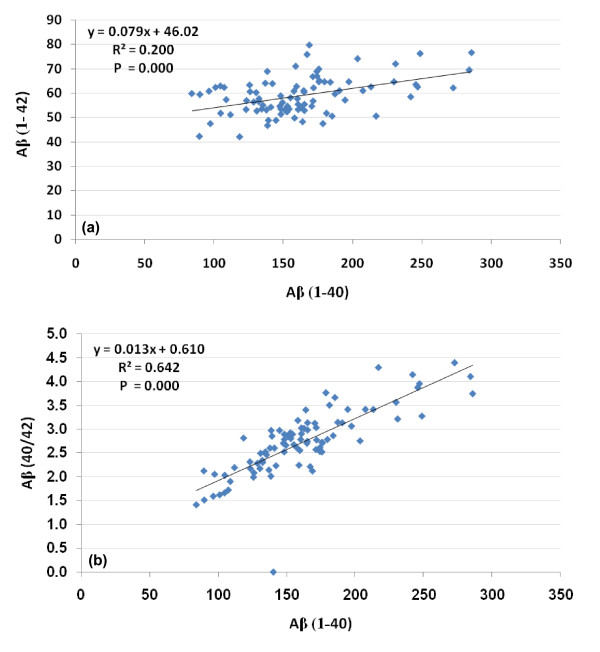
Pearson positive correlations between measured parameters with best fit line.

Table 2 and Figure 2 show the Pearson correlations between the three measured variables.

**Table 2:** Pearson correlation test between the measured amyloid beta peptides

Parameters	R (Person Correlation)	**Sig**.	
Aβ (1-40)~ Aβ (1-42)	0.447	0.000	P^a^

Aβ (1-40)~ Aβ (40/42)	0.859	0.000	P^a^

^a^: Positive correlation

**Figure 3:**
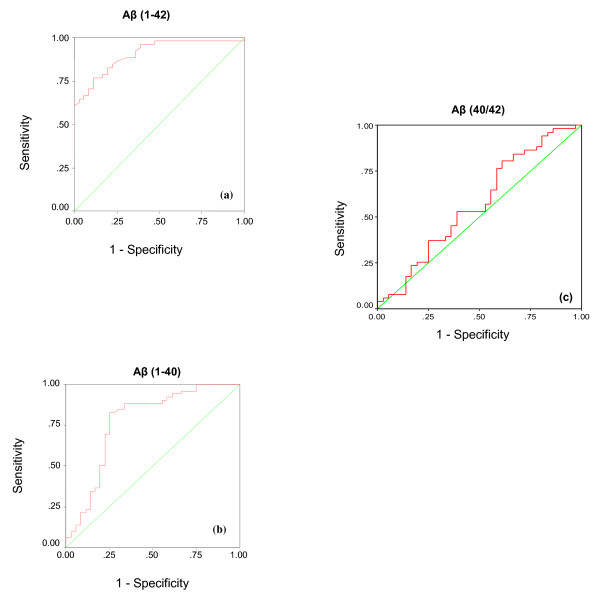
**Receiver Operating Characteristics (ROC) analysis of the measured parameters in plasma of autistic patients.** ROC curves showing area under the curves, specificity and sensitivity of Aβ (1-42) (a), Aβ (1-40) (b) together with Aβ (40/42) (c).

Table 3 and Figure 3 show the results of ROC analysis: the area under the curve (AUC) and the specificity and sensitivity of Aβ (1-40), Aβ (1-42), and Aβ (40/42).

**Table 3:** ROC analysis of the three measured amyloid beta peptides.

Parameter	Area under the curve	Best Cutoff value	Sensitivity %	Specificity %
Aβ (1-40)	0.773	165.00	82.7%	75.0%

Aβ (1-42)	0.905	60.29	82.4%	80.6%

Aβ (40/42)	0.571	3.02	80.39%	38.89%

## 4. Discussion

In general, Aβ (1-40) is less neurotoxic, less common in the neuritic plaques of AD, and less likely to be involved in the neuropathology of AD than Aβ (1-42). However, Aβ (1-42) is more difficult to study than Aβ (1-40) because of polymerization. As is the case for any peptide, the concentrations of Aβ are a balance between its rate of synthesis and its rate of degradation [26]. Moreover, it has been reported that the concentrations of Aβ in brain and blood are in equilibrium, through the blood-brain-barrier (BBB), and that peripheral sequestration of Aβ may shift this equilibrium toward the blood, eventually drawing out the excess from the brain ("sink" effect) [27].

In the present study, the concentrations of both Aβ (1-40) and Aβ (1-42) were lower in autistic children than in age-matched control subjects (Table 1 Figure 1). This finding could be attributed to loss of Aβ equilibrium between the brain and blood, which may lead to the failure to draw out Aβ from the brain, i.e., increased blood-to-brain influx and decreased brain-to-blood efflux across the BBB. The observed low plasma concentrations of Aβ (1-40) and (1-42) in the autistic Saudi children, together with the LPS hypothesis of Jaeger et al. [28], could be easily supported by the findings of many studies showing that children with autism have an overload of gram-negative bacteria that contain LPS as a causative agent of mitochondrial dysfunction, a biochemical aspect recorded in a high percentage of autistic patients [16,29-32].

Proteolytic cleavage of amyloid precursor protein (APP) by the sequential actions of β- and γ-secretases form the neurotoxic Aβ peptide, which typically consists of 40 or 42 amino acid residues (the amyloidogenic pathway). This could help to suggest the impairment of β- and γ- secretase's levels and/or kinetics in autistic patients showing lower plasma concentrations of Aβ (1-40) and Aβ (1-42). This suggestion could be supported by the work of Sokol et al. [33] and Bailey et al. [34], who reported higher plasma concentrations of secreted APPα in autistic patients than in aged-matched control subjects and their recommendation to measure sAPP-α concentrations in serum and human umbilical cord blood as a potential tool for the early diagnosis of autism.

The pathogenesis of many neurological disorders is also believed to be associated with oxidative stress, which may be responsible for the dysfunction or death of neurons. Aβ can serve as a metalloenzyme to catalyze the generation of neurotoxic H_2_O_2 _from O_2 _through binding and reduction of Cu (II) [35]. Fang et al. (2010) reported that oligomer and the fibril form of Aβ (1-42) can promote the generation of H_2_O_2 _when the concentration of co-incubated Cu (II) is below a critical level [36] and the amount of TBARS reactivity is greatest when generated by Aβ (1-42) ˃˃ Aβ (1-40) [37].

At normal physiological conditions, SOD1 is known to increase cellular resistance to oxidative stress [38]. However, when the SOD enzyme is overexpressed at levels that are much higher than those of other antioxidant enzymes, such as GPx and CAT, or higher than the ability of cells to supply reducing equivalents, increased oxidative stress is observed [39]. Oxidative damage is likely because of the generation of ˑOH from the interaction of accumulating H_2_O_2 _with redox cycling proteins via Fenton-like chemistry [40]. The lower Aβ (1-42) and Aβ (1-40) plasma concentrations reported in the present study, together with the proposed higher brain concentrations of both peptides, could be easily related to the findings of previous reports by Al-Gadani et al. (2009), which demonstrated that autistic Saudi children are under H_2_O_2 _stress because of overexpression of SOD and normal CAT activity [16].

Recent evidence suggests that the low-density lipoprotein receptor-related protein 1 (LRP1) transcytoses Aβ out of the brain across the blood-brain barrier (BBB) [41]. Deane et al. [42] reported that in RAP knockout mice the expression of LRP-1 is reduced in the brain and that Aβ (1-40) elimination from the brain to blood is also reduced. These findings provide evidence for a direct protein-protein interaction between LRP and Aβ and demonstrate that this interaction takes place in an isoform-specific manner. This finding shows that Aβ isoforms are differentially transcytosed or endocytosed through the BBB and that LRP at the BBB favors the clearance of Aβ isoforms relative to high β sheet content.

Recently, Gu et al. [43] reported that exposure to lead (Pb^2+^) increases the concentrations of Aβ in the brain and inhibits LRP1 expression; this finding could explain the suggested Aβ accumulation in the brains of the autistic Saudi children in the present study. This explanation could find support in the work of El-Ansary et al. [31], who found that Pb^2+ ^concentrations were significantly higher in the red blood cells (RBC) of 12 of 14 autistic Saudi children than in those of control subjects; this finding indicates that autistic children are more vulnerable to Pb^2+ ^toxicity and hence are more likely to accumulate Aβ (1-40) and (1-42) in their brains. This could be supported through considering the lower Aβ 40/42 ratios recorded in the present study in autistic patients compared to control subjects. It is well known that clearance and transport from brain to blood is facilitated by an increased Aβ 40/42 ratio present at young ages [44]. Moreover, young mouse model harboring a mutation favoring generation of Aβ 1-42 over Aβ 1-40 had a low Aβ 40/42 ratio, was shifted to plaque deposition [45].

Our speculated explanation could find a support in the most recent experimental study of Frackowiak et al. [46] in which they used immunoblotting to prove that frozen autopsy brain samples of 9 autistic patients show accumulation of Aβ 40 and 42 in the cerebellum and cortex. Moreover, the explained association between chronic Pb toxicity previously recorded in 15/15 autistic patients of Saudi Arabia and the speculated Aβ accumulation of the present study is in good agreement with the finding of Garcidue˜nas [47,48] which show that Children's exposure to urban air pollution increases their risk for auditory and vestibular impairment through the accumulation of Aβ 42 in their brainstems. To better understand changes in Aβ production, accumulation, and clearance in autistic patients, it will be necessary to continue studying the normal and disease-related metabolism of Aβ in various body fluids and in the brains of rodents used in animal models of autism.

Nutrition plays a vital role in the methylation of DNA, specifically the homocysteine (HCY)/S-adenosylmethionine (SAM) cycle. This cycle requires the presence of folate and B12, which facilitate the conversion of HCY to methionine, which is then converted to SAM. SAM then serves as a source of methyl groups for multiple methylation reactions, including the methylation of DNA. The increased concentrations of Aβ in the brains of autistic Saudi children could be easily explained by the hypothesis recently proposed by Lahiri and Maloney [49]. They proposed that most AD cases follow an etiology based on Latent Early-life Associated Regulation or "LEARn" as a two-hit model [50,51]. They reported that exposure to metals, nutritional imbalance (low B12), and other environmental stressors modify potential expression levels of AD-associated genes (e.g., Aβ peptide precursor protein) in a latent fashion. Autistic patients are known to exhibit oxidative stress [16], high RBC lead concentrations [31], and impaired DNA methylation because of a remarkably lower concentration of S-adenosylmethionine (SAM) [52]. On the basis of this information, the two-hit hypothesis of Lahiri and Maloney [50] could explain the impaired Aβ concentrations in the plasma of autistic Saudi children, as reported in the present study.

The Pearson correlations presented in Table 2 and Figure 2 show that while there was only an acceptable level of correlation between Aβ (1-40) and Aβ (1-42) (correlation coefficient less than 0.5), a very good level of association was found between Aβ (1-40) and Aβ (40/42) ratio (correlation coefficient of 0.859). This could be helpful to suggest that lower values of Aβ (1-40) and Ab (40/42) ratio must be recorded together as biomarker in a patient diagnosed as autistic while an association between Aβ (1-40) and Aβ (1-42) is not a must.

Table 3 and Figure 3 illustrate the results of ROC analyses of the two measured Aβ peptides. Although Aβ 40/42 ratio reported low value of sensitivity and specificity, absolute values of Aβ (1-42) and Aβ (1-40) reported satisfactory figures of sensitivity and specificity to be considered as potential biomarkers for autism.

## Abbreviations

AD: Alzheimer's disease; Aβ: Amyloid Beta; APP: amyloid precursor protein; ASD: autism spectrum disorders; BBB: Blood brain barrier; CAT: catalase; CNS: central nervous system; GPx: glutathione peroxidase; GSH: glutathione; HCY: homocysteine; H_2_O_2: _hydrogen peroxide; IQ: Intelligence quotient; LRP1: low density lipoprotein receptor-related protein 1; MIF-1: melanocyte stimulating hormone release inhibiting factor number 1; RBC: red blood cells; ROC: Receiver operating characteristics curve; ROS: Reactive oxygen species; SAM: S-adenosylmethionine; SD: Standard deviation; SOD: superoxide dismutase; SP: Senile plaques; Tyr-MIF-1: tyrosine melanocyte stimulating hormone release inhibiting factor number 1.

## Competing interests

The authors declare that they have no competing interests.

## Authors' contributions

AE designed the study and drafted the manuscript. ABB helped to draft the manuscript and performed the statistical analysis. MO helped with the English polishing. LA provided samples and participated in the design of the study. All authors have read and approved the final manuscript.
